# Rezafungin in fluconazole-resistant and refractory candidiasis: the first Brazilian experience

**DOI:** 10.1590/S1678-9946202668020

**Published:** 2026-02-16

**Authors:** Wdson Luis Lima Kruschewsky, Jeanne Aiko de Souza Nakagawa, Lumena Pereira Machado Siqueira, Mariane Taborda, Vítor Falcão de Oliveira, Edson Abdala, Adriana Satie Gonçalves Kono Magri, Marcello Mihailenko Chaves Magri

**Affiliations:** 1 Universidade de São Paulo, Faculdade de Medicina, Hospital das Clínicas, Divisão de Clínica de Moléstias Infecciosas e Parasitárias, São Paulo, São Paulo, Brazil; 2 Universidade de São Paulo, Faculdade de Medicina, Hospital das Clínicas, Divisão de Laboratório Central, Seção de Microbiologia, São Paulo, São Paulo, Brazil; 3 Universidade de São Paulo, Faculdade de Medicina, Instituto do Câncer do Estado de São Paulo, São Paulo, São Paulo, Brazil; 4 Universidade de São Paulo, Faculdade de Medicina, Instituto de Medicina Tropical de São Paulo, Laboratório de Hepatologia por Vírus (LIM-47), São Paulo, São Paulo, Brazil

**Keywords:** Rezafungin, Echinocandins, Invasive candidiasis, Esophageal candidiasis, Antifungal resistance

## Abstract

Invasive candidiasis is associated with high morbidity and mortality, and the rising of antifungal resistance underscores the need for new therapies. Rezafungin, a second-generation echinocandin, enables once-weekly dosing, achieves high plasma concentrations, and shows potent in vitro activity. We report two Brazilian cases showing its clinical utility: (i) fluconazole-resistant *Candida tropicalis* bloodstream infection in a patient with colorectal cancer and chronic kidney disease and (ii) azole-refractory *C. albicans* esophagitis in a patient with autoimmune polyglandular syndrome type 1. Both achieved rapid clinical response and microbiological clearance. These are the first documented cases of rezafungin use for invasive candidiasis in Brazil.

## INTRODUCTION

Invasive candidiasis (IC), one of the most common invasive fungal infections worldwide, is associated with substantial morbidity and mortality, the rates of which exceed 50% in Brazil^1^. Although *Candida albicans* remains the most frequently isolated species^2^, epidemiology has shifted over the past decades toward non-*albicans Candida* species, which often show reduced susceptibility or resistance to azoles^2,3^.

Echinocandins have been the first-line therapy for IC and candidemia due to their fungicidal activity, favorable safety profile, and minimal drug interactions. However, first-generation agents (caspofungin, micafungin, anidulafungin) require daily intravenous dosing, have short half-lives, and may achieve suboptimal peritoneal concentrations, limiting outpatient use^4^.

The development of rezafungin, a novel second-generation echinocandin (approved in 2023 for adults with IC and candidemia), involved modifying the anidulafungin molecule to achieve its extended half-life, enabling once-weekly dosing^4,5^. This pharmacokinetic advantage, combined with high peak plasma concentrations from front-loaded dosing and potent in vitro activity against a broad range of *Candida* spp., makes rezafungin an attractive option for both hospital and outpatient antifungal therapy^5,6^.

To ensure that these represent the first published cases of rezafungin use in Brazil, we conducted a comprehensive literature search on LILACS, SciELO, MEDLINE, PubMed, and PubMed Central. This study found no prior reports describing the clinical use of rezafungin in Brazil. Here, we report two distinct cases of candidiasis: one of fluconazole-resistant *C. tropicalis* candidemia in a patient with advanced colorectal cancer and another of azole-refractory *C. albicans* esophagitis in a patient with autoimmune polyglandular syndrome type 1 (both successfully managed with rezafungin). These cases illustrate its potential role in diverse clinical scenarios, including simplified dosing regimens, reduced hospitalization, and treatment of azole-resistant infections.

### Ethics

Written informed consent was obtained from the patients for publication of this case report and accompanying images.

## CASE REPORT 1

### Fluconazole-resistant *C. tropicalis* candidemia and intra-abdominal candidiasis in a patient with advanced colorectal cancer

A 68-year-old male with a history of rectal adenocarcinoma (treated in 2022 with laparoscopic rectosigmoidectomy and adjuvant chemotherapy) and stage IV chronic kidney disease presented with local recurrence at the colorectal anastomosis in January 2024. He underwent abdominoperineal resection with mucous fistula creation and perineal reconstruction, followed by eight additional cycles of chemotherapy. In February 2025, he developed progressive pelvic disease with fever and foul-smelling discharge from the surgical site. Empirical treatment with amoxicillin–clavulanate was initiated for presumed surgical site infection.

**Figure 1:**
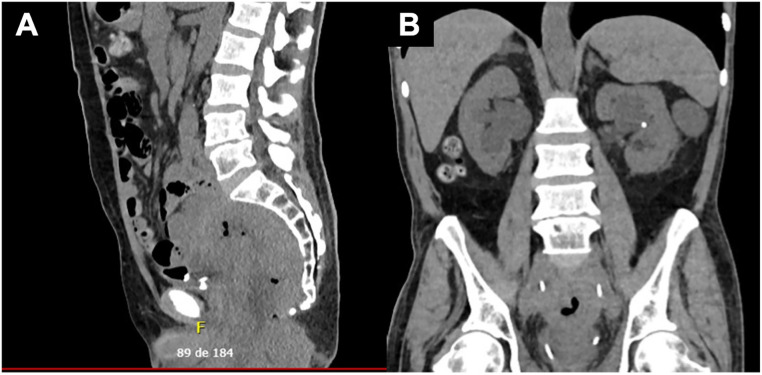
Contrast-enhanced computed tomography of the abdomen and pelvis showing an extensive pelvic mass with a large central necrotic/liquefied component (sagittal view, A) measuring about 18 cm, with fistulization to the intergluteal sulcus and anterior perineal region occupying the presacral space and causing cortical erosions. Lateral extension involves the left levator ani and contacts the left obturator internus muscle, with associated chronic thrombosis of the left internal iliac vein. Coronal view (B) shows anterior extension with invasion of the seminal vesicles and contact with the prostate, peritoneal involvement, and superior extension to the distal ureters, resulting in upstream dilatation. No free fluid or intra-abdominal collections are observed.

Two months later, he was admitted to the emergency department of a referral oncology hospital in Sao Paulo, Brazil, with severe perineal pain, local bleeding, fever, nausea, and vomiting. His peripheral blood cultures were initially negative. Laboratory testing showed leukocytosis (13,800/mm^3^; 90% neutrophils) and acute kidney injury (KDIGO stage II; serum creatinine 4.84 mg/dL). Empirical antibiotic therapy with vancomycin and piperacillin–tazobactam was initiated. Despite clinical stability, he remained febrile. Repeated blood cultures grew vancomycin-resistant *Enterococcus faecium* and fluconazole-resistant *C. tropicalis* (MIC 32 mg/L) that showed susceptibility to micafungin (MIC 0.25 mg/L). An abdominal CT scan showed an extensive pelvic mass with a large central necrotic component occupying the presacral space, with fistulization to the perineal region and local invasion of adjacent pelvic structures, including pelvic floor muscles, seminal vesicles, and distal ureters, resulting in upstream ureteral dilatation (Figure 1).

Antibiotic therapy was adjusted to linezolid, and antifungal therapy with rezafungin was initiated: 400 mg intravenously as a loading dose, followed by 200 mg one week later. The patient became afebrile after the first dose, with subsequent blood cultures negative from day two onward. Ophthalmologic examination and transthoracic echocardiography excluded endophthalmitis and infective endocarditis. The patient was discharged three weeks after receiving his first dose of rezafungin (day 23), completing therapy as an outpatient without recurrence.

## CASE REPORT 2

### Azole-refractory *C. albicans* esophagitis in autoimmune polyglandular syndrome type 1

A 34-year-old male with autoimmune polyglandular syndrome type 1 (adrenal insufficiency, growth hormone deficiency, and type 1 diabetes mellitus) had a long-standing history of recurrent oroesophageal candidiasis since childhood, frequently presenting with severe odynophagia and dysphagia requiring hospitalization for intravenous antifungal therapy. He had previously undergone prolonged outpatient courses of terbinafine, fluconazole, and itraconazole, and had received echinocandins or amphotericin B formulations during severe episodes. His most recent regimen consisted of oral itraconazole capsules 600 mg/day; yet symptoms persisted.

**Figure 2:**
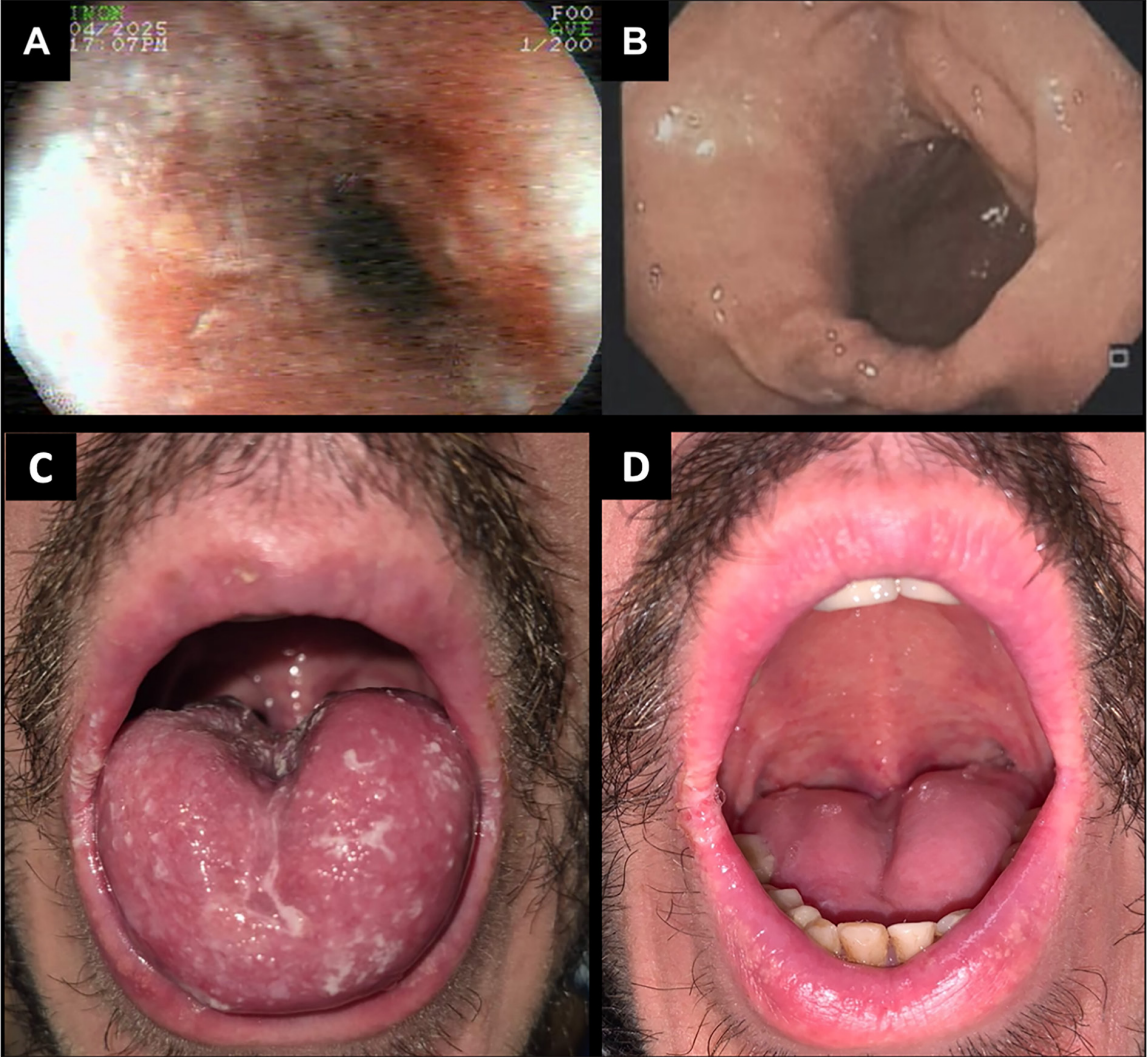
Upper gastrointestinal endoscopy and oral cavity examination before and after rezafungin treatment for azole-refractory *Candida albicans* oroesophageal candidiasis: (A) Upper gastrointestinal endoscopy before treatment showing whitish plaques and mucosal hyperemia in the distal esophagus (Kodsi grade I); (B) Follow-up endoscopy two weeks after the last rezafungin dose showing complete resolution of esophageal lesions and normalization of mucosal appearance; (C) Oral cavity examination before treatment evincing extensive pseudomembranous lesions on the dorsal surface of the tongue and palate; (D) Oral cavity examination one month after treatment showing complete resolution of lesions with normal mucosal appearance.

Figure 2 shows the comparative oral and upper gastrointestinal endoscopic findings before and after rezafungin treatment. Upper gastrointestinal endoscopy) revealed whitish esophageal plaques compatible with Kodsi grade I esophagitis (Figure 2A). Oral examination before treatment showed extensive pseudomembranous lesions involving the dorsal surface of the tongue and the palate—consistent with active oral candidiasis (Figure 2C). Histopathology and culture identified *C. albicans* resistant to fluconazole (MIC >256 mg/L) but susceptible to micafungin (MIC 0.125 mg/L). The lack of response to oral therapy and the need to avoid prolonged hospitalization required the initiation of intravenous rezafungin under a 400-mg loading dose, followed by a 200 mg dose one week later on an outpatient basis. The patient reported marked improvement in odynophagia and dysphagia within days, with resolution of oral mucosal lesions. Follow-up upper gastrointestinal endoscopy two weeks after the last dose showed complete resolution of esophageal lesions (Figure 2B). At a one-month follow-up visit, he remained asymptomatic with no clinical or endoscopic evidence of recurrence (Figure 2D).

## DISCUSSION

This report documents the first clinical use of rezafungin in Brazil, in two distinct settings: fluconazole-resistant *C. tropicalis* bloodstream infection and azole-refractory *C. albicans* esophagitis. Both cases underscore the potential clinical utility of this second-generation echinocandin in scenarios requiring prolonged therapy, rapid fungemia clearance, or outpatient parenteral antifungal treatment (OPAT).

Rezafungin is a structurally modified anidulafungin analogue that has been engineered to enhance chemical stability and extend its terminal half-life to approximately 130 h, enabling high peak plasma concentrations after a front-loaded regimen (400 mg on day 1) with sustained exposures above the MIC for ≥7 days^4,5^. Pharmacokinetic and pharmacodynamic studies have shown broad tissue distribution, with drug concentrations up to four-fold higher in key organs compared with plasma, including intra-abdominal lesions^7^. Pre-clinical models also show potent activity against mature *C. albicans* biofilms^8^ and robust AUC/MIC-driven fungicidal activity in disseminated candidiasis due to *C. tropicalis and C. dubliniensis* ^9^. In vitro, rezafungin retains activity against a broad spectrum of *Candida* spp., including non-wild-type isolates with reduced susceptibility to first-generation echinocandins and multidrug-resistant *C. auris* from multiple clades^10^.

The phase III ReSTORE trial has shown the non-inferiority of once-weekly rezafungin to daily caspofungin for global response at day 14 (59% vs 61%) in candidemia and invasive candidiasis^6^. In exploratory analyses, the median time to first negative blood culture was shorter with rezafungin (23.9 h) than with caspofungin (27.0 h)^6^. These early microbiological responses^6,11,12^ are consistent with the rapid defervescence and negative follow-up cultures in Case Report 1. The patient in Case Report 2 had previously received all other available echinocandins, which had been associated with slower symptomatic improvement and earlier recurrences.

A pooled analysis of the STRIVE and ReSTORE trials (N=294) compared once-weekly rezafungin with once-daily caspofungin for candidemia/invasive candidiasis, stratified by *Candida* species. Rezafungin proved to be an effective treatment irrespective of the baseline species. Rezafungin achieved numerically higher day-14 global cure rates than caspofungin for *C. glabrata* (71.1% vs. 60.0%) and *C. parapsilosis* (78.6% vs. 55.6%). Additionally, the rezafungin group showed numerically lower day-30 mortality rates for *C. tropicalis* (18.5% vs. 31.8%) and *C. parapsilosis* (7.1% vs. 29.6%). These advantages, which include better mycological eradication rates for *C. glabrata* and *C. tropicalis*, are attributed to its "front-loaded exposure," enabled by its increased metabolic stability and once-weekly dosing regimen^13^.

In intensive care units, Honoré et al., 2024 performed a post-hoc pooled analysis, including 113 critically ill patients. Rezafungin achieved higher mycological eradication at day five (78.3% vs 59.7%) and 14 (71.7% vs 65.7%) and a significantly shorter median time to negative blood cultures (18 h vs 38 h; P=0.001, not adjusted for multiplicity). Safety profiles were comparable, with fewer treatment-emergent adverse event discontinuations in the rezafungin group (17.4% vs 29.9%)^11^. Rezafungin may enable shorter hospital and ICU stays than daily intravenous caspofungin in patients with invasive candidiasis or candidemia, with a mean total hospital stay of 25.2 vs 28.3 days and a mean ICU stay of 16.1 vs 21.6 days, corresponding to an adjusted reduction of 4.1 ICU days (24% relative difference). Yet, these results warrant real-world confirmation^14^. These findings align with our Case Report 1 (in which rapid culture clearance facilitated earlier discharge) and 2 (in which the pharmacokinetic profile allowed effective OPAT completion). Notably, the patient in Case Report 2 had previously received all other available echinocandins (with slower symptomatic improvement and earlier relapse), further underscoring the potential clinical benefit of rezafungin in refractory disease. Although these findings require cautious interpretation, they support the hypothesis that the pharmacokinetic pharmacodynamic modeling of rezafungin can facilitate early clinical stability and potential discharge (as in our Case Report 1) and enable effective outpatient completion of therapy (as in Case Report 2). Specifically, rezafungin requires no dose adjustments for patients with hepatic impairment (including mild, moderate, or severe cases), renal insufficiency (or those receiving renal replacement therapy), or obesity (based on weight or body mass index). The consistent dosing regimen is also appropriate for older patients and those who are critically ill in intensive care units^15^.

This report has several limitations inherent to case-based descriptions. Its findings are limited to two patients, precluding any conclusions regarding the efficacy, safety, or cost-effectiveness of rezafungin in broader Brazilian populations. Both cases involved *Candida* spp. with documented azole resistance but echinocandin susceptibility. Thus, the applicability of these observations to other resistance profiles remains uncertain. Microbiological clearance was only documented via culture-based methods (that is, no molecular or biomarker-based monitoring was performed). Additionally, the contribution of concomitant antimicrobial therapy and surgical or endoscopic interventions to the favorable outcomes cannot be fully excluded. Finally, follow-up was limited to one month. Therefore, late relapses or reinfections may have been missed. Controlled clinical trials or prospective observational studies in local settings are necessary to validate these observations and to assess pharmacoeconomic impact.

Based on a health system perspective, the short-term feasibility of rezafungin use in Brazilian public hospitals remains uncertain, as the drug is yet to be incorporated into the Unified Health System. However, its pharmacokinetic profile (allowing for once-weekly dosing) may confer indirect economic advantages when compared with first-generation echinocandins, particularly by facilitating earlier hospital discharge, reducing length of stay in intensive care units, and enabling OPAT in selected patients. These potential benefits could partially offset higher acquisition costs by decreasing overall healthcare resource utilization, including daily intravenous administration, nursing workload, and prolonged hospitalization. Nevertheless, no formal pharmacoeconomic analyses evaluating rezafungin in the Brazilian public healthcare setting are currently available, and direct comparisons with existing echinocandins regarding cost-effectiveness require cautious interpretation. Prospective real-world studies and local cost-utility analyses will be essential to determine whether rezafungin can offer a favorable cost-benefit profile and sustainable implementation within public hospitals in Brazil.

## CONCLUSION

In summary, the combination of prolonged half-life, high peak drug exposure, retained potency against wild- and non-wild-type *Candida* spp., and favorable safety profile supports the role of rezafungin as a therapeutic option in invasive and refractory mucosal candidiasis. Its potential to shorten length of stay in intensive care units and hospitals and to offer OPAT in azole-resistant infections is particularly relevant in healthcare systems with limited inpatient capacity. To our knowledge, this is the first report of rezafungin use in Brazil, documenting favorable clinical, microbiological, and endoscopic outcomes in fluconazole-resistant *C. tropicalis* candidemia and azole-refractory *C. albicans* esophagitis.

## Data Availability

The anonymized dataset generated during this study is available from the corresponding author upon reasonable request.
